# Ghosts of Yellowstone: Multi-Decadal Histories of Wildlife Populations Captured by Bones on a Modern Landscape

**DOI:** 10.1371/journal.pone.0018057

**Published:** 2011-03-28

**Authors:** Joshua H. Miller

**Affiliations:** Committee on Evolutionary Biology, The University of Chicago, Chicago, Illinois, United States of America; University of Oxford, United Kingdom

## Abstract

Natural accumulations of skeletal material (death assemblages) have the potential to provide historical data on species diversity and population structure for regions lacking decades of wildlife monitoring, thereby contributing valuable baseline data for conservation and management strategies. Previous studies of the ecological and temporal resolutions of death assemblages from terrestrial large-mammal communities, however, have largely focused on broad patterns of community composition in tropical settings. Here, I expand the environmental sampling of large-mammal death assemblages into a temperate biome and explore more demanding assessments of ecological fidelity by testing their capacity to record past population fluctuations of individual species in the well-studied ungulate community of Yellowstone National Park (Yellowstone). Despite dramatic ecological changes following the 1988 wildfires and 1995 wolf re-introduction, the Yellowstone death assemblage is highly faithful to the living community in species richness and community structure. These results agree with studies of tropical death assemblages and establish the broad capability of vertebrate remains to provide high-quality ecological data from disparate ecosystems and biomes. Importantly, the Yellowstone death assemblage also correctly identifies species that changed significantly in abundance over the last 20 to ∼80 years and the directions of those shifts (including local invasions and extinctions). The relative frequency of fresh versus weathered bones for individual species is also consistent with documented trends in living population sizes. Radiocarbon dating verifies the historical source of bones from *Equus caballus* (horse): a functionally extinct species. Bone surveys are a broadly valuable tool for obtaining population trends and baseline shifts over decadal-to-centennial timescales.

## Introduction

Animal communities worldwide are experiencing ecological perturbations due to combinations of rapid climate change, habitat destruction, and over-exploitation [1]–[3]. The paucity of multi-decadal, multi-generational studies of biological systems limits our ability to accurately track ecological changes and evaluate the magnitude and significance of recent disturbances [1], [4]–[6]. For the majority of animal communities, historical ecological data are patchy at best, limiting our ability to establish conservation and remediation goals that incorporate dynamics from past decades and centuries [1]. Vertebrate skeletal remains exposed on present-day landscape surfaces are naturally time-averaged accumulations of multiple generations and are thus unique archives of ecological history. Utilizing such death assemblages, however, requires confidence that historical patterns are not unduly overprinted by the postmortem mixing of generations or by differential preservation of species. Here, I test the ability of death assemblages to capture complex natural and human-driven changes in the populations of key species, using the well-studied history of ungulate populations within the Northern Range of Yellowstone National Park (Yellowstone), USA.

Previous studies have found that mammalian death assemblages can capture the composition and structure of the source community, and that the averaging of skeletal input across multiple generations can be advantageous for detecting historical shifts in composition [7]–[16]. Equally critical for establishing conservation goals and management directives, however, is information on how the abundances of key species have changed historically, under both natural and human-influenced environmental regimes [17]–[19]. Thus, given the rarity of long-term monitoring in most regions, there is a global need for new methods to detect the long-term trajectories of individual species. While death assemblages seemingly possess the temporal perspective needed for this work, previous tests of death assemblage fidelity to living populations have focused on community-wide representation and have not directly examined how bone accumulations record population histories of individual species. Moreover, ecological analyses of death assemblages are rare and, in the case of terrestrial large-mammals, are restricted to tropical African localities [7], [13], [20], [21], where relatively high postmortem rates of bone destruction (bones generally do not survive on the surface past the first two decades postmortem [13], [22]) limit the potential blurring effects of skeletal time-averaging. Regions in temperate and arctic latitudes, where bone decay rates are lower [23], may provide more temporally expansive data, though the impacts of increased time-averaging on a death assemblage's ecological fidelity have gone untested. These limits on current understanding of death assemblage formation and their ecological fidelity have slowed the adoption of bone surveys as a means of acquiring historical insights on large-mammal communities.

In this study I evaluate *(i)* whether death assemblages in temperate settings capture the community richness and overall structure with the same accuracy as in tropical settings, and *(ii)* the fidelity with which population changes are recorded for individual species. Naturally occurring bone accumulations are largely unexplored and undocumented, yet are likely ubiquitous across the globe. If death assemblages can be established as accurate recorders of historical diversity and population data for a range of biomes and environments, then ecological analysis of these historic resources would be a powerful complement to wildlife monitoring practices as well as isotopic and ancient-DNA analyses that can reveal changes in a population's diet, home range, and genetic history [24]–[26].

### The study system

**Figure 1:**
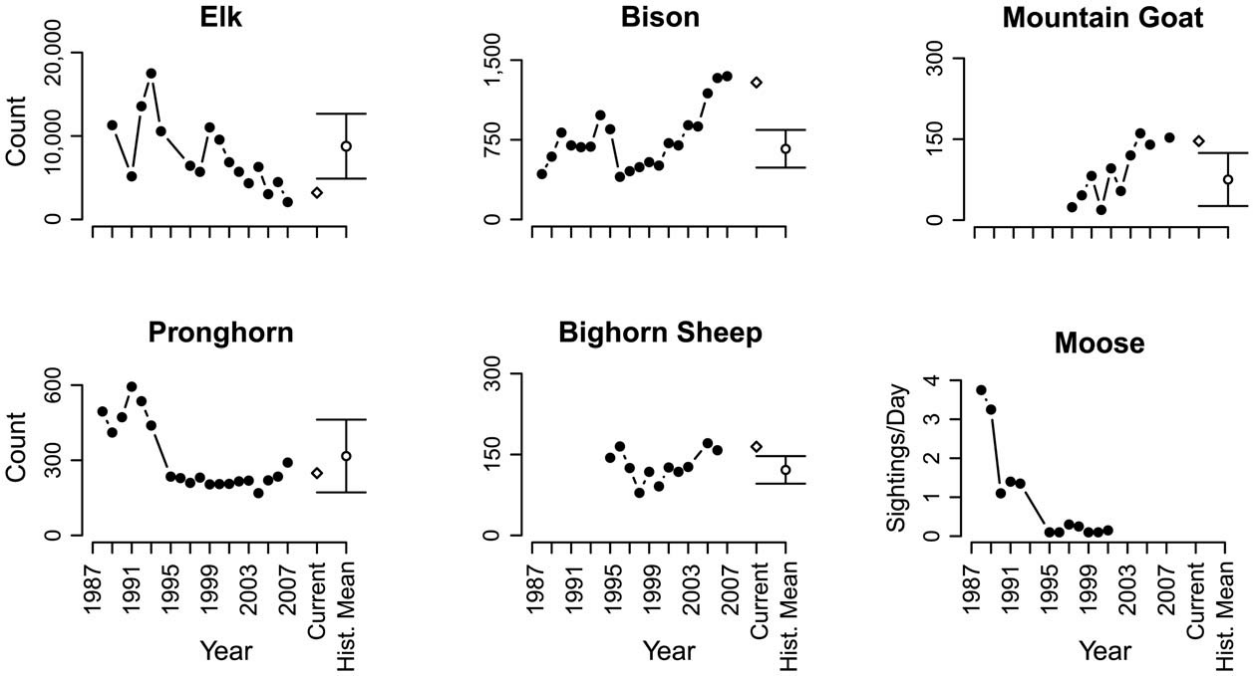
Variation in populations of six ungulate species across the Northern Range within Yellowstone National Park (Yellowstone). Data based on 11 to 21 years of aerial surveys (black circles – data from [28]–[37]). Current average population counts during the 2005–2007 study of bone assemblages are indicated by white diamonds. Mean and one standard deviation of historical surveys for each species (equal weighting of all available pre-2005 surveys) are indicated by white circle and error bars. Since 1995 (the initiation of bighorn surveys), elk populations have declined significantly (negative slope of log-transformed abundance regressed on time; p = 0.0087), while populations of bison and mountain goat (which first appeared in Yellowstone in the 1990's [42]) increased significantly (p_Bison_ = 0.00071, p_Mtn. Goat_ = 0.0083). Trends for other species are not significant (see text). Bison abundances are corrected for culls conducted by Yellowstone (see Methods, Supporting Information S1 text S2.0). Historically, moose populations have been estimated using various on-ground proxies; data plotted here are based on observations along one trail in western Yellowstone [30]. Moose surveys are no longer conducted. For details on species, see text and Supporting Information S1 text S1.0, S2.0.

Ungulate populations of the Northern Range, an important wintering area that extends beyond Yellowstone's boundaries [27], have been studied for decades, with population sizes and demography determined using aerial surveys for elk (*Cervus elaphus*), bison (*Bison bison*), pronghorn (*Antilocapra americana*), bighorn sheep (*Ovis canadensis*), mountain goat (*Oreamnos americanus*), and moose (*Alces alces*) ([28]–[37], Supporting Information S1 text S1.0; body sizes - Table S1). Populations of mule deer (*Odocoileus hemionus*) are also surveyed, but herd sizes and population trends within the study area itself are less well understood [27], [37], [38], and so deer are excluded from this study. Overall, aerial surveys of Yellowstone ungulates show dramatic changes over the last twenty years (Figure 1) including significant reductions in moose following the 1988 wildfires [30], [39], [40], major declines in elk following the 1995 reintroduction of wolves [29], [40], recent dramatic increases in bison [29], [40], and the first appearance of mountain goats to the Yellowstone ecosystem in the 1990's [41], [42]. In addition, pronghorn populations experienced significant declines in the early 1990's from which they have been slowly recovering [34]. Bighorn sheep populations have also partially recovered from a significant disease-related population reduction in the 1980's [33], [43], though populations were steady overall between 1995 and 2007. Finally, although not a member of the native community, horse (*Equus caballus*) was the dominant mode of transportation for cavalry and early tourism from the mid-1880's until the 1910's and 1920's, when the cavalry was removed and roads leading up to and within Yellowstone increasingly permitted access by motorized vehicles [44], [45]. In subsequent decades, horse use has been very limited, essentially eliminating this species as a contributor to the death assemblage. This known history of Yellowstone ungulates presents an ideal system for testing the ability of time-averaged death assemblages to record the composition and species-level dynamics of temperate terrestrial ecosystems on decadal to centennial scales.

The Yellowstone death assemblage was surveyed using a series of forty 1 km transects distributed equally among four generalized habitats: rolling grassland, river-margin, swamp- and lake-margin, and forest (survey methods adapted from [7]; see Methods for details). All encountered bones and bone fragments were documented for location, species identity, skeletal element type, and bone weathering stage (a proxy for postmortem duration [22]). The Minimum Number of Individuals (MNI) was calculated for each transect based on species, ontogenetic age, skeletal element, and weathering stage (following [7], [13], [20]) and compared to the living abundance data from aerial surveys (Figure 1, Supporting Information S1 text S1.0, Figure S1). Because bone surveys required three years of fieldwork (2005–2007), aerial survey data (corrected for the removal of culled bison; Supporting Information S1 text S2.0) were averaged over the same time-interval, referred to as the “current” living community (Figure 1).

## Results and Discussion

**Figure 2:**
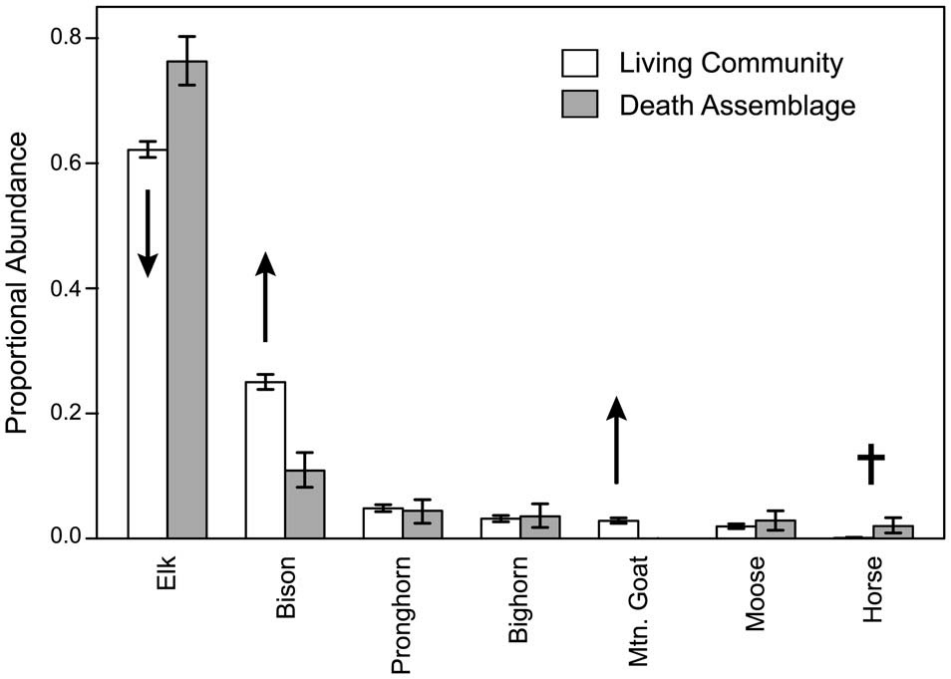
Proportional abundance of ungulate species in the current (2005–2007) living community compared with abundances in the naturally occurring death (bone) assemblage. Death assemblage composition is based on pooling bone counts across all transects and habitats (total MNI  = 451). Error bars are bootstrapped 95% confidence intervals. Arrows show direction of all significant population trends for species within the last decade (1995–2007; see Figure 1). Cross denotes extirpated species (horse) with respect to the death assemblage. The ranked abundance of species in the death assemblage closely resembles the ranking of these same species in the living community (Spearman rho  = 0.89, p = 0.0123). Significant live-dead mismatch is found in two native species – elk and bison – as well as in the recent invading species (mountain goat) and the extirpated species (horse). The direction of each mismatch is correctly predicted by known population changes: recently increasing species are disproportionally rare in the death assemblage, while species with documented population declines are disproportionally abundant – the ghosts of past larger populations.

The ungulate death assemblage closely resembles the current living ungulate community in richness and composition (Figure 2). The death assemblage contains all native ungulate species and captures the relative abundance structure of the entire community (rank-order correlation test, Spearman rho  = 0.89, p = 0.0123; sample-size-corrected Bray-Curtis similarity  = 0.82, 95% CI: 0.78–0.86). Bootstrapped 95% confidence intervals on the proportional abundances of each species indicate that elk and horse are significantly over-represented in the death assemblage relative to the current Yellowstone ungulate community (*i.e.*, skeletal ghosts of past larger populations), and that bison is significantly under-represented, consistent with its formerly smaller population size (Figure 1, Figure 2). The only large ungulate not sampled from the death assemblage is mountain goat, which first appeared in the ecosystem in the early 1990's [42].

Such high live-dead agreement in proportional abundances is remarkable, though observed differences for some species warrant further investigation into possible biases in the death assemblage. Three commonly-recognized sources of bias in death assemblages are *(i)* over-representation of large-bodied species [20], [46], *(ii)* over-representation of heavily predated species [47]–[49], and *(iii)* over-representation of species having short generation times [20], [50], [51]. The over-abundance of elk and horse in the Yellowstone death assemblage is consistent with a preservational bias favoring larger body-sizes (360 kg and 450 kg respectively, Table S1), but other species comparisons contradict such a bias. For example, bison (the largest animal in the ecosystem – 700 kg) is strongly *under*-represented in the death assemblage, and the smallest ungulates in Yellowstone (pronghorn: 50 kg and bighorn: 80 kg) are equally abundant in both the death assemblage and the living community, contrary to expected under-representation. The over-abundance of elk is also consistent with known bias in wolf diet; a database of known ungulates killed by wolves since 1995 indicate that elk is their primary prey on the Northern Range (>90% of wolf diet [40]). Elk are not significantly preyed upon by other species within Yellowstone (27). My bone transects intersected 23 ungulates (all elk) known to have been killed by wolves (Supporting Information S1 text S3.0). Removing these carcasses from the analysis does not change the results (Figure S2), and thus abundance patterns in the death assemblage are not significantly biased by non-uniform predation. Finally, Yellowstone ungulate species are quite similar in fecundity (all producing 1–2 young per year) and life expectancy (all reaching between one and two decades [27], [52]) so differences in population turnover are unlikely to skew the proportional representation of species. Rejecting these potential biases as controls on live-dead deviations in proportional abundances suggest that alternative mechanisms are responsible – possibly those derived from historical events within Yellowstone.

### The Yellowstone death assemblage faithfully represents historical populations

**Figure 3:**
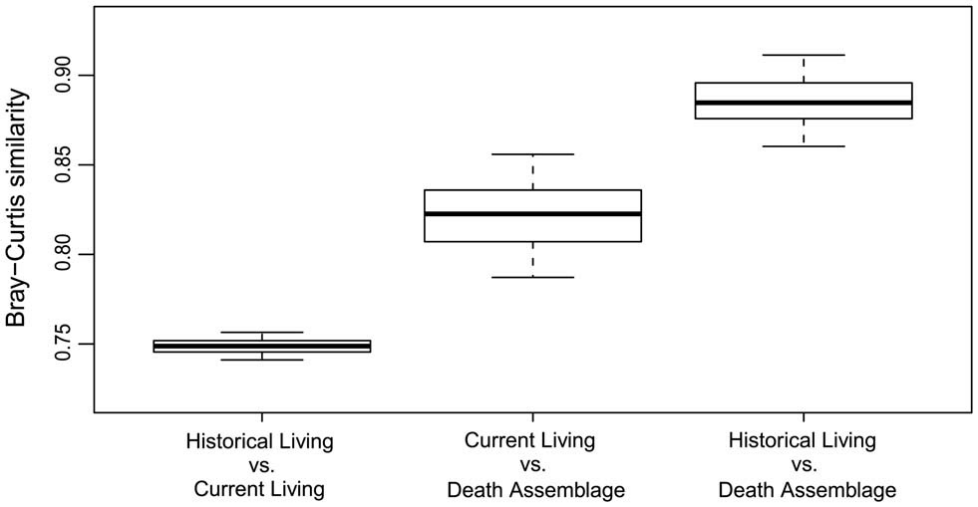
Boxplots of Bray-Curtis similarity values for all pair-wise comparisons of three types of sample-size standardized community data: the current living community, the historically averaged living community, and the death assemblage. Bray-Curtis similarity increases with increasing similarity in the temporal span of the data being compared. The composition of the death assemblage is very similar to the historical living community, and thus provides a better approximation of historical populations than do surveys of the living community today. Boxes display median and interquartile range (whiskers show 95% confidence intervals) of paired-comparisons between 10,000 iterations of a bootstrapped sample-size standardization routine.

Because the Yellowstone death assemblage is time-averaged (containing skeletal remains from past generations), differences in proportional abundances between the living and dead data might signify that the death assemblage has not yet equilibrated to recent strong population changes in the living community [15], [53], [54]. If so, then the composition of the Yellowstone death assemblage should resemble historically averaged census data (pre-2005) more closely than it does the “current” (2005–2007) living community. Testing this prediction, I find nearly perfect agreement between the death assemblage and the multi-decadal averaged historical composition of the living community (spearman rho  = 0.93, p<0.01, sample-size-corrected Bray-Curtis Similarity = 0.89, 95% CI: 0.86–0.92, Figure 3). This agreement surpasses that between the death assemblage and the current living community (values in Figure 2 caption, Figure 3), and between the current living community and averaged *historical* survey data (spearman rho  = 0.89, p<0.05; sample-size-corrected Bray-Curtis Similarity  = 0.75, 95% CI: 0.74–0.76, Figure 3). The death assemblage provides a better approximation of historical populations than do surveys of the living community today.

Furthermore, each significant deviation between the death assemblage and the current living community involves a species whose current populations *(i)* deviate significantly (greater than one standard deviation; Figure 1) from historical means, and *(ii)* have experienced significant population expansions or contractions within the last decade (measured as the slope of log-transformed abundance regressed on time since 1995, when surveys consistent with other ungulates were instituted for bighorn; [55], [56], Figure 1). Moreover, live-dead offset in the proportional abundances of these species (Figure 2) is always in the direction predicted by known population change (Figure 1): over-abundance in the death assemblage for species with contracting populations (elk; significance of population trend, p = 0.0087), and under-abundance for expanding species (bison, mountain goat; p_Bison_ = 0.00071, p_Mtn. Goat_ = 0.0083). In the case of mountain goats, the death assemblage is likely exhibiting a temporal lag: the small populations of this recently arrived species have yet to measurably contribute to the death assemblage (Supporting Information S1 text S3.0). Populations of bighorn (which do not show live-dead offset) are currently higher than their decadal-mean, but their populations have fluctuated widely since consistent surveying began in 1995. While they have shown population growth during the early 2000's [43], bighorn do not show evidence of a trending population across the available time series (p = 0.48). Pronghorn, whose populations are also equally represented in the death assemblage and current living community, similarly show no significant temporal trend in living population size between 1995 and 2007 (p = 0.62). For horse, the death assemblage retains an appropriately faint ecological memory of a species that has not contributed skeletal remains for many decades.

Accelerator Mass Spectrometry radiocarbon dating of bone collagen verifies that horse bones in the Yellowstone death assemblage are residual elements from distant generations (three individuals date to 135+/−35, 135+/−35, and 140+/−30 radiocarbon years; Table S2). These results also enrich a limited sample of radiocarbon-dated large-mammal remains from modern land surfaces (primarily from arctic latitudes [57], [58]) that support the concept of prolonged time-averaging in natural bone accumulations.

### Bone weathering distributions illustrate past population reductions

**Figure 4:**
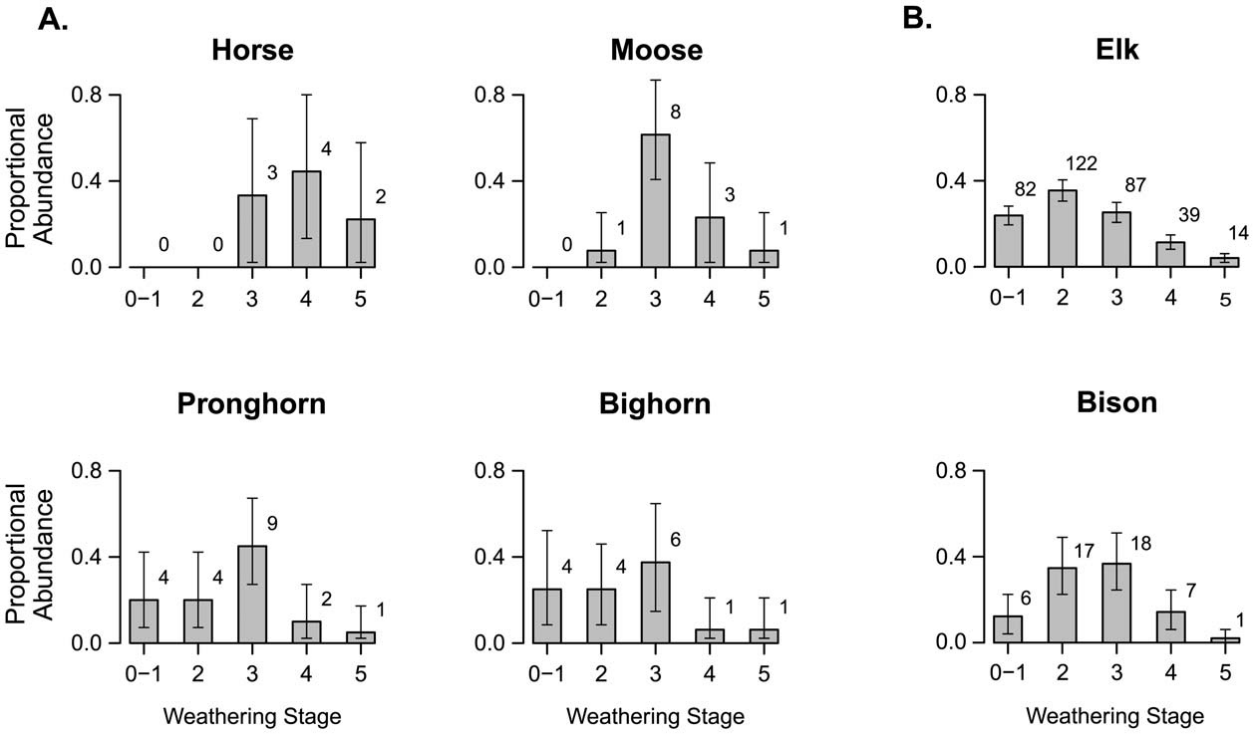
Frequency distributions of bone weathering stages (WS) for Yellowstone ungulates. The death assemblage faithfully documents species' population histories including those that experienced (A) sharp historical declines in population size and (B) recent and less severe or punctuated population changes. Numbers associated with bars are counts of individuals (MNI), error bars are bootstrapped 95% confidence intervals. For historical population data, see Figure 1; bighorn population reduction occurred in 1982 [43]. The newly arrived mountain goat was not encountered in the death assemblage. Weathering Stage values (0 to 5) represent increasing degrees of weathering as a function of postmortem exposure (scale adapted from [22]). Yellowstone bones remain in WS0 for brief periods of time, typically less than a year, and thus individuals composed of such bones are pooled with individuals in WS1 (representing up to ∼6 years postmortem in Yellowstone) to increase sample size and better approximate cohorts of death. Historical drops in populations are evident in the relative frequency of bone weathering stages. For example, the locally extirpated species (horse), which has had negligible input of bones since the 1920's, has no individuals in the initial WS categories. Species whose populations declined within the 15–20 years prior to the 2005–2007 bone survey (moose, pronghorn) are represented by bones that include fresh input but MNI are mostly in WS3; these are ghosts of past higher rates of bone input (higher populations). The peak bighorn frequency in WS3 is not significant: on this cold-temperate landscape, bones generated >25 years ago have lower survival rates, making it difficult to discern older fluctuations in populations size, such as the severe 1982 bighorn population crash. Recent increases in bison populations have been obscured by removal of culled animals (see text).

Historical population data contained in death assemblages are also evident in weathering stages (WS) of the bones themselves. Bones progress through well-documented, easily distinguishable physical stages of weathering as a function of the duration of postmortem exposure [22]. Working with ungulate bones in a semi-arid tropical setting, Behrensmeyer [22] showed that bones stay in WS0 and WS1 (unweathered and lightly weathered) for up to the first few years postmortem before continuing their progression to WS2 (incorporating bones within the first postmortem decade), WS3 (incorporating bones within and beyond the first postmortem decade), and WS4 and WS5 (maximum survival durations have yet to be fully calibrated, but reach 30+ years at least [13]). This calibration provides some expectations for linking frequency distributions of species' weathering stages with abundance changes over time, though temperate systems have extended overall weathering durations compared to tropical settings [23]. Yellowstone horse bones are thus expected to be primarily in fairly advanced weathering stages if they are indeed remnants of early 20^th^ century populations. As predicted, the frequency distribution of weathering stages for horse bones (MNI) in Yellowstone is skewed towards high values (≥WS3; Figure 4).

Consistent patterns between known-population changes and the frequency distributions of bone weathering stages are found in all species. For example, the majority of skeletal elk individuals (major population decline within the last decade) are in WS2 (Figure 4). In addition, the majority of moose and pronghorn individuals (both experienced dramatic population declines between the late 1980's to early 1990's) are most frequently in the more advanced WS3. Bighorn skeletons show a broader peak of specimens in WS2 and WS3, suggesting a fading record of the extreme population reduction of 1982 [43]. Owing to vagaries of decay and burial among bones on a natural landscape and over such a long period of time (>25 years), the signal of this sharp demographic event is somewhat damped in the death assemblage, with a flatter peak in the WS frequency distribution than seen in species having more recent sharp population declines.

Thus, controls on bone survival at the landscape surface impose limits on the historical reach of death assemblage information. Under the temperate weathering regime of Yellowstone, strong population reductions occurring within 15–20 years of a bone survey are quite distinct in the death assemblage, whereas older strong pulses of mortality will tend to become obscured. The Yellowstone death assemblage also illustrates that large-scale harvesting, poaching, or culling of a population can degrade the legibility of a WS frequency distribution. Culling of Yellowstone bison in the late 1990's through early 2000's, for example, decreased the numbers of individuals entering the natural death assemblage during that interval and has inflated the proportion of older, weathered individuals (the broad peak in WS2 and WS3; Figure 4), despite the increase in population during the decade preceding bone surveys.

### Bone surveys perform well in disparate climatic and ecological settings

Previous work with terrestrial large-mammal death assemblages in tropical Africa (Amboseli, Kenya [13]) showed excellent recovery of ungulate species in the death assemblage (see also [8], [9]) and moderately- to highly-positive rank-order abundance correlations to the source community from different study periods over 40 years (Spearman rho's = 0.69_1964–1969_, 0.73_1970–1976_, 0.62_1993–1998_, 0.89_1999–2004_). The temperate Yellowstone death assemblage (Spearman rho  = 0.89_Current Living_, 0.93_Historical Living_) meets or exceeds the data recovery from this tropical ecosystem. Such congruence in rank-order fidelity is striking given the large differences in climate between the two regions as well as species richness (Amboseli: S = 15, Yellowstone: S = 7) and ranges of body-size (Amboseli: 20–4,000 kg, Yellowstone: 50–700 kg). While the ability of *tropical* death assemblages to record population shifts of individual species remains to be further tested (though some tracking of relative abundance changes is implicit in the good community-level results found in Amboseli across multiple decades), the agreement of the two studies indicates that surface bone accumulations may be a globally useful proxy for placing large-mammal communities in recent and historical contexts. Furthermore, extended durations of bone survival in temperate (and arctic) environments make them particularly well-suited for obtaining ecological insight for historical periods from which monitoring data is often problematic or absent. This suggests that robust historical population data may still be recoverable on species across large regions of North America, Eurasia, and elsewhere that lack reliable historical data and/or that remain poorly studied.

### Conclusions

The results of this study – the first controlled taphonomic evaluation of species abundance and temporal resolution of large-mammal death assemblages in temperate settings – confirm and expand a source of biodiversity data important for both paleontologists and biologists. For paleontologists, the high fidelity of the Yellowstone death assemblage to the source community strengthens confidence in the ecological information recovered from deeper-time fossil deposits, which is critical to understanding extinct ecosystems and using them to test for general principles of ecological dynamics. For biologists, surveys of naturally accumulated and time-averaged skeletal remains provide a non-invasive and relatively inexpensive and rapid means of acquiring a decadal-scale historical perspective on population changes for key mammal species in regions where scientific data are scarce, problematic, or lacking entirely. Deviations in species presence-absence, rank-abundance, and proportional abundance between time-averaged bone assemblages and present-day surveys of living populations can reveal past shifts in population structure arising from both natural and anthropogenic causes. Frequency distributions of bone weathering stages can provide independent data on past population states of individual species and their fluctuations over multiple decades and beyond. Landscape-scale surveys of death assemblages, especially when combined with census data of the living community, can provide a uniquely powerful means of recognizing ecological change over time-scales that are usually unavailable to biological surveys. Particularly informative for regions with limited wildlife monitoring records, this approach provides a critical new tool for establishing elusive baseline data for rapidly changing ecosystems.

## Methods

The Yellowstone death assemblage was surveyed using forty 1 km transects. Transects were distributed equally among four generalized habitats: rolling grassland, river-margin, swamp- and lake-margin, and forest (survey methods adapted from [7]). Transect widths were habitat/visibility dependent. Grassland transects were 100 m wide (50 m on either side of a midline), forest transects were 60 m wide (30 m on either side of a midline), and river- and lake-margin transects extended 30 m shoreward from the water's edge. Transects were spaced a minimum of 1 km apart to limit the possibility that bones from one individual would be sampled multiple times due to scavenger dispersal of one carcass' bones. Transects were walked by myself and two field assistants who sampled either side of transect lines (or beside one another along water margins) and flagged all observed bones, bone fragments, and carnivore feces. I collected all data on each bone occurrence using standardized data sheets including location (Garmin eTrex Vista or Rhino II WAS enabled GPS), species identity, skeletal element type, ontogenetic age, and bone weathering stage (a proxy for postmortem duration [22]). Specimens were identified in the field using reference atlases or collected and identified with the use of comparative collections at the Field Museum, Chicago, IL and Montana Fish Wildlife and Parks, Bozeman, MT. Transects were sampled during the summers of 2005, 2006, and 2007. The Minimum Number of Individuals (MNI) was calculated for each transect based on species, ontogenetic age, skeletal element, and weathering stage (following [7], [13], [20]) and compared to the living abundance data from aerial surveys (Figure 1). Due to extensive bison culling conducted by Yellowstone staff, which removed potential bison from the death assemblage, annual herd sizes were corrected to account for these losses before comparison (Supporting Information S1 text S2.0). To match the duration over which bone surveys were conducted (2005–2007), aerial survey data were averaged over the same time-interval, referred to as the “current” living community (Figure 1, Figure 2). Both the death assemblage and the “current” living community were also compared to multi-decadal averages of the historical survey data (all available data from 1987 through 2004; Figure 1, Figure 3). Tests for significant positive or negative population trends between 1995 and 2007 were conducted by regressing log-transformed abundance data from aerial surveys on time [55], [56]. The initiation of bighorn surveys consistent with other species defined the start-date of these regressions.

To compare the Yellowstone death assemblage to the living ungulate community, MNI from the bone assemblages were converted to proportional abundances and compared to proportional abundance values derived from surveys of the living populations. The fidelity of the ecological data available in the Yellowstone death assemblage was analyzed by comparing metrics of community composition and structure; relative abundance between datasets was tested using the Spearman rank-order correlation ([59]; providing a fidelity measure of rank-order abundance) and sample-size standardized bray-curtis similarity ([60]; providing a fidelity measure that is sensitive to the proportional abundances of species). Rank-order correlations on data from Amboseli [13] were restricted to mammals (*i.e.*, excluded ostrich).

To test the representation of individual species' abundances between the death assemblage and the living community, 95% bootstrapped confidence intervals were calculated [59] for each species' proportional abundance and compared between the living (survey data) and dead (bone MNI) representations of that species. All statistical analyses were conducted in the open source statistical language R (R Core Development Team, version 2.9.2).

To radiometrically-age bone samples, collagen was extracted (following [61]) and subjected to Accelerator Mass Spectrometry (AMS) analyses. All preparations and AMS radiocarbon dating were conducted at the Center for Mass Spectrometry; Lawrence Livermore National Labs, CA.

## Supporting Information

Figure S1 **Study area (gray stipple) within the Northern Range of Yellowstone National Park (Yellowstone).** Although aerial population surveys take place throughout the Northern Range (inside and north of Yellowstone borders), the living abundance data used here are restricted to individuals recorded where the death assemblage was sampled.
(TIF)

Figure S2 **Rank-order and proportional abundance of the current living populations (as in** **Figure 2** **) compared to the Yellowstone ungulate death assemblage after correction for potential predation bias (wolf-killed ungulates).** Spearman coefficient (rho  = 0.89, p = 0.0123) remains unchanged and all significant and non-significant relationships between the living community and its death assemblage remain the same. The death assemblage is not significantly biased by non-uniform predation.
(TIF)

Table S1 (DOC)

Table S2 (DOC)

Supporting Information S1 (DOC)
