# Supplementary figures and images for: Ghosts of Yellowstone: Multi-Decadal Histories of Wildlife Populations Captured by Bones on a Modern Landscape
Source: PLoS One. 2011 Mar 28;6(3):e18057. doi: 10.1371/journal.pone.0018057 (PMC3065453; doi:10.1371/journal.pone.0018057)

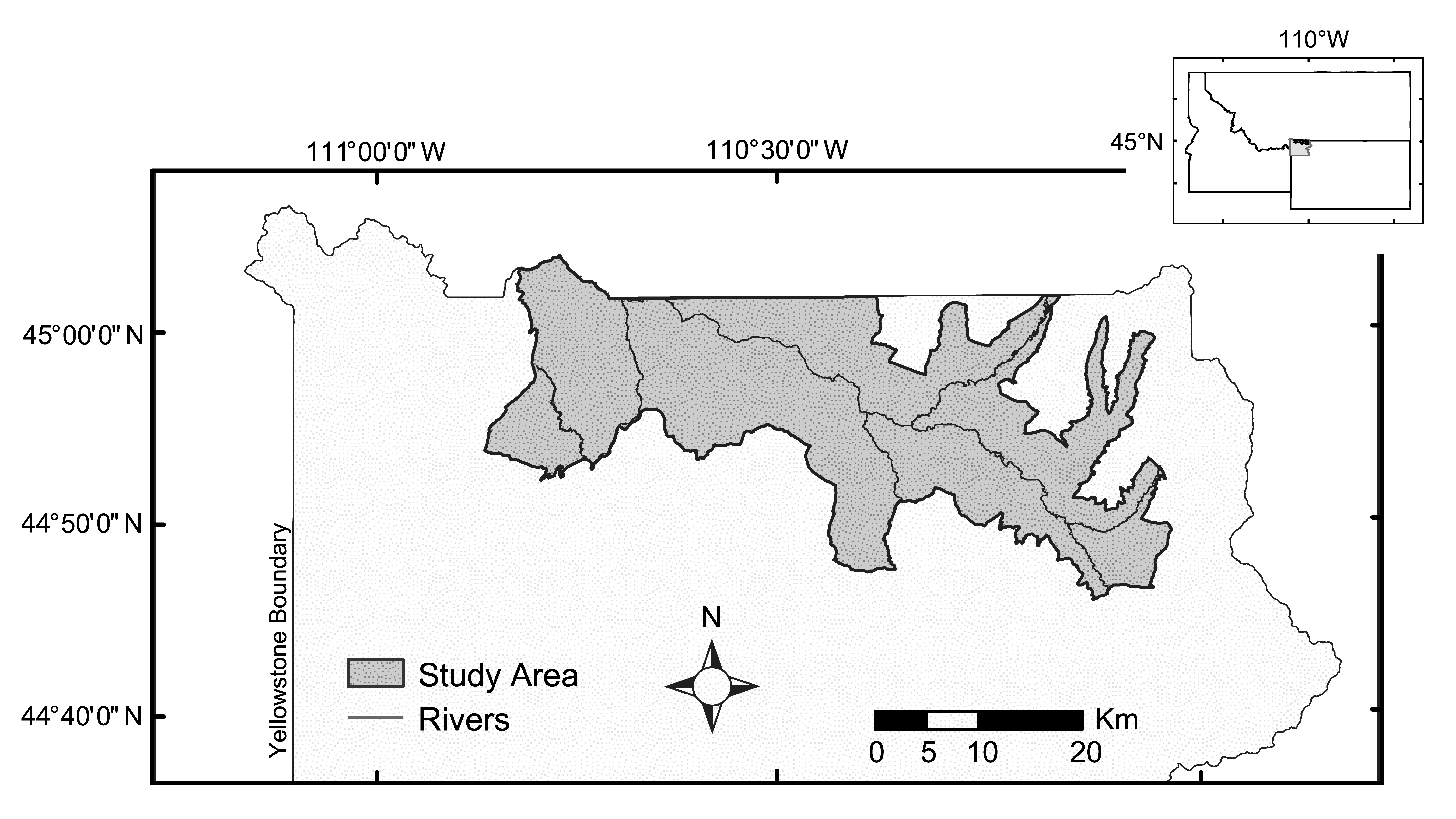

Supplement: Figure S1 — Study area (gray stipple) within the Northern Range of Yellowstone National Park (Yellowstone). Although aerial population surveys take place throughout the Northern Range (inside and north of Yellowstone borders), the living abundance data used here are restricted to individuals recorded where the death assemblage was sampled. (TIF) [file pone.0018057.s001.tif]

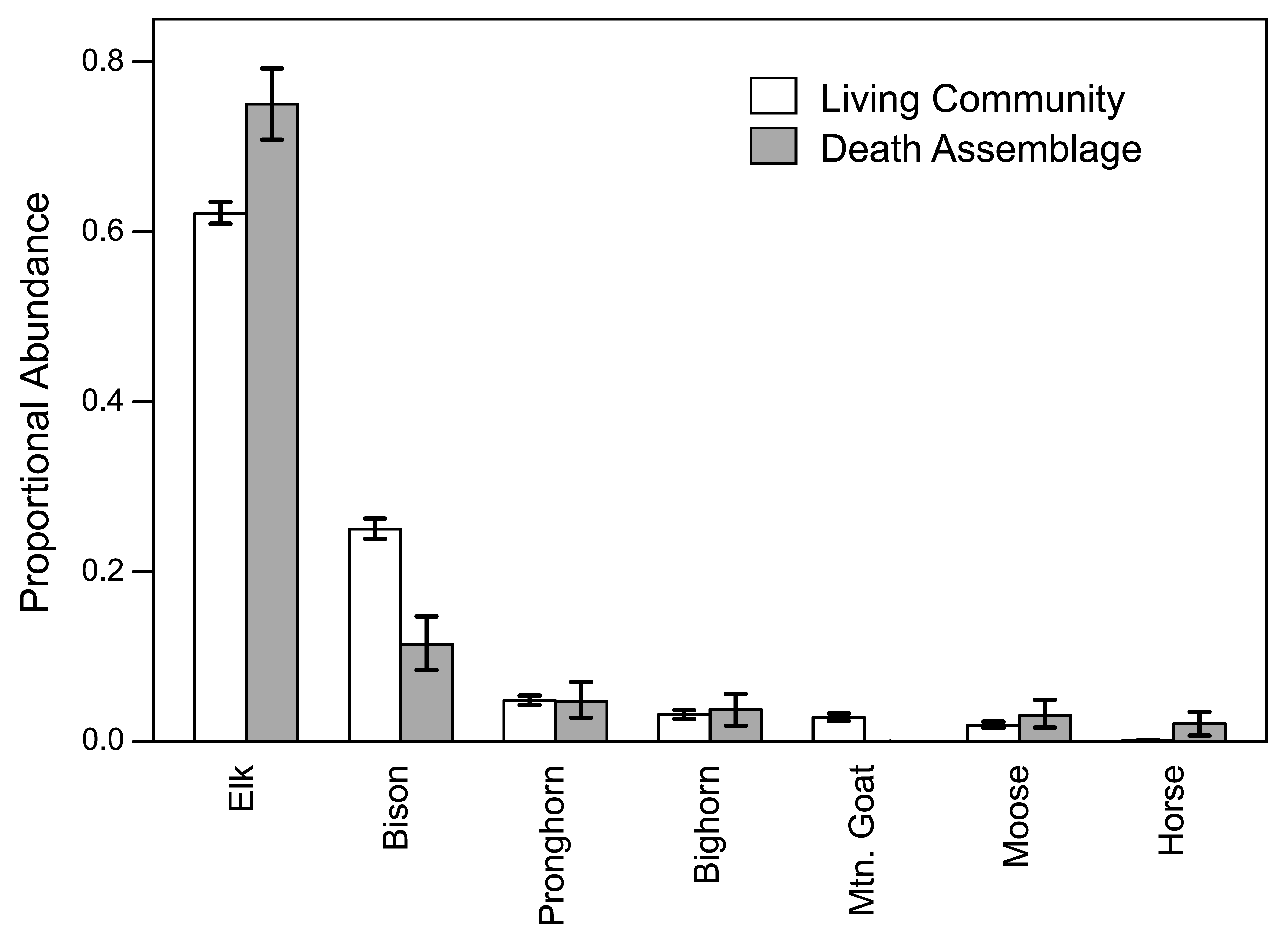

Supplement: Figure S2 — Rank-order and proportional abundance of the current living populations (as in Figure 2 ) compared to the Yellowstone ungulate death assemblage after correction for potential predation bias (wolf-killed ungulates). Spearman coefficient (rho = 0.89, p = 0.0123) remains unchanged and all significant and non-significant relationships between the living community and its death assemblage remain the same. The death assemblage is not significantly biased by non-uniform predation. (TIF) [file pone.0018057.s002.tif]
