# Supplementary material for: Ghosts of Yellowstone: Multi-Decadal Histories of Wildlife Populations Captured by Bones on a Modern Landscape
Source: PLoS One. 2011 Mar 28;6(3):e18057. doi: 10.1371/journal.pone.0018057 (PMC3065453; doi:10.1371/journal.pone.0018057)
Supplement: Table S1 — (DOC) [file pone.0018057.s003.doc]

**Table S1. Survey counts and body-size data for living and dead samples of the Yellowstone ungulate community.**

|  | **Elk** | **Bison** | **Pronghorn** | **Bighorn** | **Mt. Goat** | **Moose** | **Horse** |
| --- | --- | --- | --- | --- | --- | --- | --- |
| **Death Assemblage** | 344 | 49 | 20 | 16 | 0 | 13 | 9 |
| **Current Living** | 3206 | 1290 | 249 | 164 | 146 | 100 | 5 |
| **Historical Living** | 7734 | 759 | 306 | 129 | 51 | 130 | 5 |
| **Body-size (kg)** | 360 | 700 | 50 | 80 | 100 | 600 | 450 |

“Death Assemblage” counts are MNI results from the bone surveys (2005-2007). "Current Living" abundances are averaged across all aerial surveys of the living species conducted during the same interval (2005-2007) within the Northern Range study area. “Historical Living” abundances are means of available aerial survey data on living species from 1987 through 2004. Estimated horse abundance used here is significantly larger than the numbers that actually die annually with YNP, and so sets a high (conservative) bar to assess the representation of horse within the death assemblage (see SI; text S1.0 and S2.0). Body-size data from [18].
