# Supplementary material for: Ghosts of Yellowstone: Multi-Decadal Histories of Wildlife Populations Captured by Bones on a Modern Landscape
Source: PLoS One. 2011 Mar 28;6(3):e18057. doi: 10.1371/journal.pone.0018057 (PMC3065453; doi:10.1371/journal.pone.0018057)
Supplement: Table S2 — (DOC) [file pone.0018057.s004.doc]

**Table S2. Accelerator Mass Spectrometry ^14^C-dates on bone collagen of horse bones from the Yellowstone death assemblage.**

| Yellowstone Catalogue Number | Specimen ID | Element | 13C fraction of modern | Error (+/-) | 14C age | Error (+/-) |
| --- | --- | --- | --- | --- | --- | --- |
| YELL 194717 | T01-11-06 08 | Metacarpal | 0.9834 | 0.0038 | 135 | 35 |
| YELL 194718 | T01-15-06 100 | Femur | 0.9836 | 0.0038 | 135 | 35 |
| YELL 126114 | T01-16-06 09 | Metacarpal | 0.9826 | 0.0033 | 140 | 30 |

Dates were obtained from the Center for Accelerator Mass Spectrometry (Lawrence Livermore National Labs, CA) using standard collagen extraction techniques [23]. These dates illustrate the broad time over which ecological data are available in temperate death assemblages.
