# Supplementary material for: Ghosts of Yellowstone: Multi-Decadal Histories of Wildlife Populations Captured by Bones on a Modern Landscape
Source: PLoS One. 2011 Mar 28;6(3):e18057. doi: 10.1371/journal.pone.0018057 (PMC3065453; doi:10.1371/journal.pone.0018057)
Supplement: Supporting Information S1 — (DOC) [file pone.0018057.s005.doc]

**Supporting Information Text**

**S1.0 Data on the Living Yellowstone Ungulate Community.**

Aerial population surveys of the Yellowstone National Park (Yellowstone) ungulates are conducted by the Northern Yellowstone Cooperative Wildlife Working Group (NYCWWG) and Yellowstone staff along standard geographic routes [1] and are species-specific (separate flights are dedicated for elk, bison, pronghorn, bighorn sheep, and mountain goat). Moose are not currently monitored (see discussion below).

Weather conditions, funding, and other variables prevent surveys of each species every year. The most robust and consistent survey data for the ungulate community are from 1987 to present. To ensure data quality across years, elk surveys from 1988 and 1990 are not included in my live-dead comparisons because they were conducted in poor weather and are probably under-estimates of true population sizes [2]. Annual surveys of bighorn and mountain goats were initiated in 1995 and 1997, respectively.

The NYCWWG survey data include ungulate populations outside as well as inside Yellowstone boundaries. Only data for ungulate populations inside Yellowstone were used in analyses here, in order to match the geographic coverage of my death assemblages data (Figure S1). Surveys of mountain goats extend approximately one kilometer outside of Yellowstone, and data on bighorn used here include surveys of a narrow stretch of land from the Yellowstone boundary to Cinnabar Mountain (just north of the Yellowstone border). Bighorn populations show significant migration around and outside Yellowstone and show characteristics of a single metapopulation [3, 4]. Surveys of sheep extending beyond Cinnabar Mountain are not included.

**Moose.** Moose are notoriously difficult to study in Yellowstone due to their low abundance and solitary life habit [5, 6]. While hundreds of moose were known to inhabit Yellowstone in the first half of the twentieth century [5], surveys have never been considered quantitatively robust. Moose abundance has generally been monitored using indices instead of raw counts. The moose index provided in Figure 1 (“Moose”) is based on the number of moose observed per day while on horseback along a particular trail in the western portion of Yellowstone [5]. All recorded moose indices show a large population reduction following the 1988 wildfires [5, 6]. Current populations are not actively monitored, but they are thought to be only one-quarter [5] of pre-fire populations, which were estimated at 400 individuals [7]. Thus, current moose populations are modeled here as 100 individuals.

**S2.0 Estimating skeletal input from bison and horse**

**Bison culls and expected bison abundance in the death assemblage.** Bison in Yellowstone are subjected to periodic culls during winter months, largely to minimize the transmission risk of *Brucellosis* to nearby cattle herds [8]. When culled, bison are sent to a slaughterhouse, thereby removing them as potential input to the death assemblage. Thus, surveyed bison within Yellowstone overestimate the number available to the death assemblage in a given year. To estimate the population of bison that is actually available to the death assemblage, annual bison culls from the northern areas of Yellowstone are subtracted from northern herd population surveys. Complicating the issue, Yellowstone bison form two groups: the central herd, and the northern herd [9]. Recent work has shown significant migration from the central herd to the northern herd during winter months [10-12]. Migrations of the central herd appear to depend on herd size, and large populations of the last decade may have been particularly amenable to such movements. Thus, during times when the central herd is very large, many of the animals culled from the north are actually members of the central herd. Without accommodation for these migrating (and immediately-culled) individuals, directly subtracting the culled numbers from the northern herd’s surveyed abundances would produce inappropriately low estimates of bison available to the death assemblage in a given year. The unsuitability of this correction method is particularly apparent when numbers of culled individuals from the north nearly equal or surpass estimates of total northern herd size [9]. Data on the herd identity of culled individuals would inform the number of northern herd bison that are removed from the population, but those data are not systematically recorded. In lieu of these data, expert information (Rick Wallen, Yellowstone Bison Biologist) can establish the proportion of northern culls that are actually of northern herd individuals, enabling more appropriate corrections of the number of bison available to the Northern Range death assemblage. Annual cull numbers subtracted from annual surveys of the northern herd are adjusted as follows: no subtraction for 2003 and 2004 (all culls were of central herd animals), one third of total culls in 2005 (two-thirds were of central herd animals), and fifty percent of culls for 2006 and 2007. From 1987-2002, bison were culled during only six winters (including culls of very few individuals). To correct the expected death assemblage for these events, it is assumed that culled animals were distributed equally between the two herds. These adjustments provide the best estimates of bison actually available to the Northern Range death assemblage. Furthermore, the results on rank-order fidelity and all live-dead offsets as observed in Figures 2 and S2 are found with or without these cull-corrections. Additionally, the same results are also found after over-correcting for culled bison by directly subtracting culled northern individuals from northern herd abundances (creating the inappropriately small population estimates of living bison described above). Herd sizes and cull numbers were acquired from the literature [9, 11, 13, 14].

**Horse deaths in Yellowstone.** Horses from mounted Yellowstone rangers or small touring groups can occasionally introduce their skeletal remains into the Yellowstone death assemblage, but these deaths are rare. Yellowstone does not formally document horse deaths within the Park, but interviews with Yellowstone biologists and rangers revealed that nine horses were known to have died within Park boundaries (including areas outside the study area) between 1990 and 2007 (A. Mitchell, pers comm.). Furthermore, Yellowstone often tries to recover these carcasses, move them to centralized carcass dumps, or disperse them with dynamite, which further limits the potential of these rare deaths contributing to the death assemblage. To model the expected annual horse input to the Yellowstone death assemblage, a known-overestimate of five individuals was used. This was designed to set a high bar for the death assemblage: if the death assemblage produces more than this over-estimate, horse abundance in the death assemblage can be considered a significant anomaly relative to the current ungulate community.

**S3.0 Discussion**

**Removing the effects of wolf-predation on the death assemblage.** Since the reintroduction of wolves to Yellowstone in 1995, the Wolf Management Division of Yellowstone has maintained a database of all known animals killed by wolves [15, 16]. Within Yellowstone, this dietary catalogue is probably particularly complete for the Northern Range, where visibility is generally excellent and monitoring efforts are consistently high. Using the Wolf Management database, the wolf-generated portion of the death assemblage sample can be identified and excluded from the death assemblage by removing all kills whose locations intersect with the transects of the bone surveys. This is accomplished by overlaying the bone transect polygons with the complete wolf-kill database (ESRI ArcGIS, v.9.2) and identifying wolf-killed carcasses that intersect those transects. The conservative assumption is made that all wolf-killed carcasses that intercept transects were found during the bone surveys. Thus, to correct for wolf-bias, the total number of wolf-killed individuals for each species was subtracted from the pooled sample of the death assemblage.

**Mountain goats.** The absence of mountain goats in the surveyed death assemblage might have several explanations. For example, given the high fidelity of the death assemblage to all other Yellowstone ungulates, the absence of mountain goat might reflect their recent arrival to the ecosystem – that is, a lag could exist between when a species arrives on a landscape and when it appears in the death assemblage, owing to small initial population size and long individual life-spans relative to initial duration of occupation. Alternatively, their absence in the death assemblage might reflect highly-specific habitat requirements. Bone transects intersected only with the lower edges of the steep hillside terrains often preferred by mountain goats [17].

Wildlife studies indicate that mountain goats inhabit many mountain “islands” throughout Yellowstone [17] and that individuals must cross intervening lowland areas. Because mountain goats may be more susceptible to predation in such non-ideal areas [18], probabilities of mortality may be higher during these treks. Many of these pathways were directly surveyed by bone transects. Thus, the lack of strong overlap between bone transects and the preferred, upland habitat of mountain goats probably does not explain their absence in the death assemblage. For example, bighorn also inhabit steep hillsides [1, 19], but were recovered frequently in the death assemblage. The most likely explanation for the absence of mountain goats in bone surveys is thus a taphonomic lag [20-22] between the first-appearance of a species in the local living assemblage and its consistent appearance in the death assemblage.

**Supporting References**

1. Houston DB (1982) The Northern Yellowstone Elk: Ecology and management. New York: Macmillan. 474 p.

2. White PJ (2009) Northern Yellowstone Cooperative Wildlife Working Group 2009 annual report (October 1, 2008-September 30, 2009). Mammoth: Northern Yellowstone Cooperative Wildlife Working Group. 29 p.

3. Keating KA (1982) Population ecology of Rocky Mountain bighorn sheep in the Upper Yellowstone River Drainage, Montana/Wyoming. MSc Thesis. Bozeman: Montana State University. 79 p.

4. Ostovar K (1998) Impacts of human activity on bighorn sheep in Yellowstone National Park. MSc Thesis. Bozeman: Montana State University. 78 p.

5. Tyers DB (2006) Moose population history on the Northern Yellowstone Winter Range. Alces 42: 133-149.

6. Tyers DB, Irby LR (1995) Shiras moose winter habitat use in the Upper Yellowstone River Valley prior to and after the 1988 fires. Alces 31: 35-43.

7. Mack JA, Singer FJ (1992) Predicted effects of wolf predation on Northern Yellowstone elk, mule deer, and moose using POP-II models. In: Varley JD, Brewster WG, editors. Wolves for Yellowstone? A report to the United States Congress. Vol. IV. Mammoth: National Parks Service. pp. 44-69.

8. NRC (1998) Brucellosis in the Greater Yellowstone Area. Washington DC: National Academy Press. 186 p.

9. Gates CC, Stelfox B, Muhly T, Chowns T, Hudson RJ (2005) The ecology of bison movements and distribution in and beyond Yellowstone National Park: A critical review with implications for winter use and transboundary population management. Calgary: University of Calgary. 313 p.

10. Garrott RA, Bruggeman JE, Becker MS, Kalinowski ST, White PJ (2007) Evaluating prey switching in wolf-ungulate systems. Ecological Applications 17: 1588-1597.

11. Bruggeman JE, White PJ, Garrott RA, & Watson FGR (2009) Partial migration in central Yellowstone bison. In: Garrott RA, White PJ, Watson FGR, editors. The ecology of large mammals in central Yellowstone: Sixteen years of integrated field studies. New York: Academic Press. pp. 217-235.

12. Geremia C, White PJ, Wallen RL, Watson FGR, Treanor JJ et al. (2011) Predicting Bison migration out of Yellowstone National Park using bayesian models. PLoS ONE 6(2): e16848. doi:10.1371/journal.pone.0016848

13. Wallen RL (2006) Interagency Bison Management Plan summary statistics for 2006. Mammoth: National Parks Service. 1 p.

14. Wallen RL (2007) Interagency Bison Management Plan summary statistics for 2007. Mammoth: National Parks Service. 1 p.

15. Smith DW, Drummer TD, Murphy KM, Guernsey DS, Evans SB (2004) Winter prey selection and estimation of wolf kill rates in Yellowstone National Park, 1995-2000. Journal of Wildlife Management 68: 153-166.

16. Smith DW, Peterson RO, Houston DB (2003) Yellowstone after wolves. Bioscience 53:330-340.

17. Lemke TO (2004) Origin, expansion, and status of mountain goats in Yellowstone National Park. *Wildlife Soc Bull* 32(2):532-541.

18. Feldhamer GA, Thompson BC, & Champan JA eds (2003) *Wild mammals of North America: Biology, management, and conservation* (Johns Hopkins University Press, Baltimore), 2nd Ed, p 1232.

19. Yellowstone National Park (1997) Yellowstone's Northern Range: Complexity and change in a wildland ecosystem. (National Parks Service, Mammoth, WY) p 148.

20. Kidwell SM (2007) Discordance between living and death assemblages as evidence for anthropogenic ecological change. *Proc Natl Acad Sci USA* 104(45):17701-17706.

21. Kidwell SM (2008) Ecological fidelity of open marine molluscan death assemblages: Effects of post-mortem transportation, shelf health, and taphonomic inertia. *Lethaia* 41(3):199-217.

22. Terry RC (2010) The dead do not lie: using skeletal remains for rapid assessment of historical small-mammal community baselines. *Proc R Soc Lond B Biol Sci* 277(1685):1193-1201.

23. Brown TA, Nelson DE, Vogel JS, & Southon JR (1988) Improved collagen extraction by modified longin method. *Radiocarbon* 30(2):171-177.
